# Single-component organic solar cells—Perspective on the importance of chemical precision in conjugated block copolymers

**DOI:** 10.3389/fchem.2023.1326131

**Published:** 2023-12-12

**Authors:** Dries Theunissen, Sander Smeets, Wouter Maes

**Affiliations:** ^1^Design and Synthesis of Organic Semiconductors, Institute for Materials Research (IMO-IMOMEC), Hasselt University, Diepenbeek, Belgium; ^2^Associated Lab IMOMEC, IMEC, Diepenbeek, Belgium; ^3^Energyville, Genk, Belgium

**Keywords:** organic photovoltaics, one-component active layer, industrial figure of merit, conjugated block copolymers, structural defects, continuous flow

## Abstract

Organic photovoltaics (OPV) present a promising thin-film solar cell technology with particular benefits in terms of weight, aesthetics, transparency, and cost. However, despite being studied intensively since the mid 90's, OPV has not entered the mass consumer market yet. Although the efficiency gap with other thin-film photovoltaics has largely been overcome, active layer stability and performance reproducibility issues have not been fully resolved. State-of-the-art OPV devices employ a physical mixture of electron donor and acceptor molecules in a bulk heterojunction active layer. These blends are prone to morphological changes, leading to performance losses over time. On the other hand, in “single-component” organic solar cells, the donor and acceptor constituents are chemically connected within a single material, preventing demixing and thereby enhancing device stability. Novel single-component materials affording reasonably high solar cell efficiencies and improved lifetimes have recently emerged. In particular, the combination of donor and acceptor structures in conjugated block copolymers (CBCs) presents an exciting approach. Nevertheless, the current CBCs are poorly defined from a structural point of view, while synthetic protocols remain unoptimized. More controlled synthesis followed by proper structural analysis of CBCs is, however, essential to develop rational structure-property-device relations and to drive the field forward. In this perspective, we provide a short overview of the state-of-the-art in single-component organic solar cells prepared from CBCs, reflect on their troublesome characterization and the importance of chemical precision in these structures, give some recommendations, and discuss the potential impact of these aspects on the field.

## 1 Introduction

Over the last decades, different types of photovoltaic (PV) modules have been developed to harness solar energy, with silicon panels dominating the market. Among the thin-film PV technologies, particularly attractive for more specialized applications which require minimal weight, flexibility, and/or (semi)transparency, organic photovoltaics (OPV) stand out in terms of “tunability”. The photoactive organic materials can be tailored to the desired application, e.g., optimal indoor or solar light absorption, color or transparency, etc., up to a level that can simply not be achieved by other technologies. Moreover, organic solar cells can be produced by large-scale printing techniques on diverse surfaces. Furthermore, inherent to their organic nature, no scarce elements are present in the key photoactive components, an important advantage with respect to sustainability (Yao and Hou, 2022; Zhang et al., 2022; Rehman et al., 2023).

Despite these benefits and promises, and the interesting prototypes provided by companies like ASCA, Heliatek, Héole, and Epishine, organic solar cells have not seen a (mass) commercial breakthrough yet. Various reasons for this can be formulated. In the early days of the field, the power conversion efficiency (PCE) of organic solar cells seriously lagged behind. On top of that, the bankruptcy of Konarka (in 2012), one of the enterprises focusing on OPV commercialization, discouraged other start-ups and potential investors. Afterwards, when the 10% efficiency limit was finally overcome, the rise of competing technologies with a spectacular growing path in terms of efficiency—notably perovskite PV—reduced the interest in OPV, both from the industrial and academic side. Luckily, some academics persevered and kept on working on new materials and fundamental aspects of OPV. This has led to an impressive jump in PCE, up to almost 20% for single-junction devices to date (Liu K. et al., 2023; Wang et al., 2023). This can largely be attributed to the emergence of powerful non-fullerene acceptors (NFAs), with a number of important benefits compared to traditional fullerenes (e.g., complementary near-infrared absorptivity and reduced energy losses) (Wang et al., 2022; Zhou et al., 2022). This efficiency boost has caused renewed enthusiasm which, combined with some skepticism on lead-based perovskite solar cells, may lead to an OPV revival.

Nevertheless, some hurdles remain and require dedicated attention. A first important matter is the operational stability under outdoor conditions (Duan and Uddin, 2020; Park S. et al., 2020; Wang et al., 2020). Besides photostability issues of the active organic components, the need of a driving force to split excitons—a result of the low permittivity of organics—rests on the intimate (nanoscale) mixing of two different materials, i.e., an electron donor (D) and acceptor (A) material. However, the bulk heterojunction (BHJ) morphology resulting from the solution deposition of the two organic materials is commonly thermodynamically unstable. Over time, external stress factors inherent to the solar cell operation (i.e., heat and illumination) stimulate unfavorable phase separation. A second important facet hindering commercialization is the lack of reproducibility in device performance. This can partly be related to the variation in molar mass distribution and a varying level of structural defects in the state-of-the-art push-pull conjugated polymers. While continuous flow chemistry enables to address the molar mass issue (Beckers et al., 2022; Smeets et al., 2023), it has not fully been embraced by the community, and the aspect of structural defects has been largely neglected, despite some recent efforts (Pirotte et al., 2018; Ma et al., 2022; Smeets et al., 2023; Vanderspikken et al., 2023).

To overcome the BHJ instability, OPV scientists envisaged the combination of the separate D and A molecules into one single material. Initial efforts on these so-called “single-component” organic solar cells (SC-OSCs) mostly focused on the “double cable” approach, wherein D-type conjugated polymers were decorated with pendant fullerene moieties (He et al., 2021, 2022; Zhang et al., 2022; Li et al., 2023). The rise of high-performance NFAs and the polymerized versions thereof [i.e., polymerized small molecule acceptors or PSMAs (Du et al., 2021; Kataria et al., 2022; Yue et al., 2022)] boosted OPV performance and opened up a new avenue for another attractive class of SC-OSCs based on conjugated block copolymers (CBCs). Solar cells prepared from these CBCs combine efficiencies up to 15% with enhanced long-term stability and simplified active layer preparation (He et al., 2021, 2022; Wu et al., 2022; Cheng et al., 2023; Li et al., 2023; Liu B. et al., 2023; Zheng et al., 2023). Self-assembly of CBCs into ordered microstructures avoids aggregation of the individual D or A materials, which allows deposition of ready-to-use inks via various coating techniques (Cheng et al., 2023; Liu B. et al., 2023; Zheng et al., 2023), opening the door for large-area OPV applications. However, additional steps need to be taken in terms of precise control over and analysis of the CBC composition (*vide infra*), which essentially defines charge generation and transport. Thereto, this perspective presents the state-of-the-art in CBC synthesis, discusses on the difficulties faced in their preparation, purification, and characterization, suggests potential solutions, and emphasizes the importance of chemical precision as a leverage for further development of the SC-OSC and the wider OPV field.

## 2 Conjugated block copolymers

**Figure 1:**
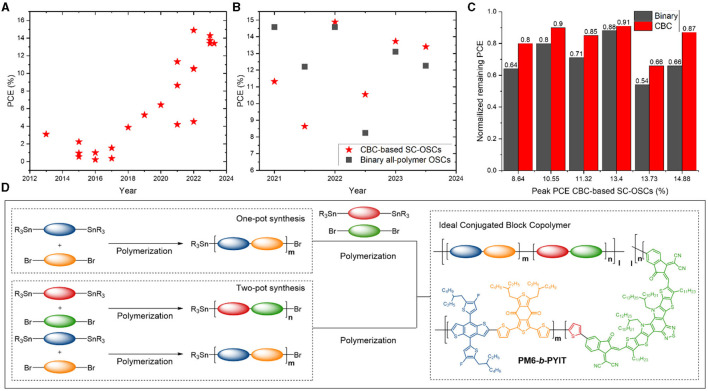
**(A)** Evolution of the power conversion efficiency of SC-OSCs based on CBCs (data in Supplementary Table 1). **(B)** Efficiency comparison of CBC-based SC-OSCs and their binary all-polymer counterparts (data in Supplementary Table 2). **(C)** Stability comparison for the CBC-based and all-polymer solar cells from **(B)** upon long-time exposure to illumination and thermal stress (details in Supplementary Table 2). **(D)** Illustration of CBC preparation via one- and two-pot synthesis pathways and chemical structure of the prototype material PM6-*b*-PYIT.

The first CBC material (P3HT-*b*-PFTBT) affording a reasonable PCE (3.1%) in SC-OSCs was reported in 2013 (Guo et al., 2013). Now, over 10 years and numerous reports later, an impressive rise in efficiency is observed (Figure 1A), facilitated by important parallel developments in the BHJ OPV field [i.e., the surge of high-performance PSMAs (Du et al., 2021; Kataria et al., 2022) and D polymers such as PM6 (Zhang et al., 2015) and D18 (Liu et al., 2020)]. In 2022, Wu et al. (2022) synthesized a CBC based on PM6 and a Y-series PSMA (PYIT). The resulting material (PM6-*b*-PYIT) (Figure 1D) yielded the most efficient CBC-based SC-OSCs to date (14.9% PCE). In terms of peak efficiency, state-of-the-art binary polymer:NFA (>19%) (Liu K. et al., 2023; Wang et al., 2023) and all-polymer OSCs (>18%) (Bi et al., 2023) still outperform SC-OSCs. However, CBC-based solar cells are quickly catching up and show untapped potential (Li et al., 2021; Wu et al., 2021, 2022; Cheng et al., 2023; Zheng et al., 2023). In some cases, SC-OSCs have even been shown to outperform their binary all-polymer counterparts (Figure 1B). Furthermore, as expected and confirmed by several studies, they often show better long-term stability (Figure 1C). This combination of high efficiency and stability results in an enhanced industrial figure of merit (i-FOM; combining the PCE, photostability, and “synthetic complexity”) for SC-OSCs (He et al., 2022), illustrating the economic relevance of this approach.

The chemical structure of a CBC can be generally described as an alternating arrangement of conjugated D and A fragments along the polymer's main chain (Figure 1D) (He et al., 2021; Li et al., 2023). They are commonly produced via a stepwise synthesis protocol using the palladium-catalyzed Stille cross-coupling to link electron-rich (“push”) and electron-deficient (“pull”) monomers as well as the D and A blocks together. Different strategies exist to achieve the desired conjugated co-block polymeric material (Li et al., 2023). Frequently, the D block is created first through Stille polymerization. By limiting the reaction time, relatively short chains with active (bromide and/or stannyl) chain ends are formed. Next, a purification step may follow, e.g., precipitation in methanol and subsequent Soxhlet extractions to remove (very) low-molar-mass chains, catalysts, ligands, and any unreacted monomer. In a next step, the individual A monomers are added to the active D polymer chains under the same conditions to incorporate the A blocks. The inverse approach, i.e., preparation of the active A block first and addition of the D monomers afterward, has been reported by Tseng et al. (2022). They postulated that the higher solubility of the A block would ensure a more homogenous polymerization of the second (D) block.

In practice, a simplified approach without purification step is used more often (Park et al., 2019; Park S. et al., 2020; Li et al., 2021, 2022; Wu et al., 2021; Liu B. et al., 2023; Zheng et al., 2023). In this one-pot synthesis (Figure 1D), the A monomers are introduced (after a short reaction time) into the reaction mixture containing the D oligomer/polymer blocks and the reaction is simply continued under the same conditions. The absence of the intermediate purification simplifies the synthesis but results in a limited control over the length of the second block and the entire structure. To gain more control over the size of both blocks, a two-pot synthesis was introduced, in which the D and A segments are prepared in separate reactions (Figure 1D). Subsequent combination of the two reaction mixtures allows for the integration of the freshly prepared active D and A chains into a CBC. Cheng et al. (2023) recently reported the synthesis of a CBC (D18-*b*-PYIT) using both one- and two-pot methods. Besides reduced solar cell efficiency, a lower PYIT content was observed for the one-pot product. These findings, in combination with the expected improved chain length control, suggest the two-pot procedure to be more adequate (Phan et al., 2022; Wu et al., 2022; Cheng et al., 2023; Li et al., 2023).

The standard conjugated polymer purification methods are applied to CBCs as well, i.e., precipitation of the crude material in methanol followed by Soxhlet extractions with methanol, acetone, hexanes, and (a) suitable chlorinated solvent(s) (Lo et al., 2021). Considering the limited purification and the applied synthesis protocols, it is only reasonable to assume that individual D or A polymers or oligomers are present in the mixture of polymer chains (Liu B. et al., 2023). Moreover, the length of the blocks, their ratio, the exact sequence (diblock, triblock, etc.), and potential gradient topology can be different for each individual polymer chain. On top of that, structural defects such as homocouplings (i.e., sequences in the polymer where two identical monomers have coupled together rather than undergoing a cross-coupling) may occur during the individual D and A Stille polymerizations and when coupling the two blocks (Hendriks et al., 2014; Vangerven et al., 2015; Lombeck et al., 2016; Pirotte et al., 2018; Smeets et al., 2023; Vanderspikken et al., 2023). In any case, a significant discrepancy between the actual structure and the simple representation of a pure diblock copolymer (as in Figure 1D) is expected. This coexistence of other species does not necessarily reflect badly on the solar cell performance. It has for instance been demonstrated that the presence of individual D and A polymers (Liu B. et al., 2023) and NFA dopants (Wu et al., 2022) can enhance device stability. However, it does impede the establishment of rational CBC structural guidelines.

Although the materials chemists responsible for the synthesis are surely aware of the structural issues, statements on the CBC composition in literature are mostly short or vague. It has to be said though that it is actually very difficult to elucidate the exact structural composition, taking in mind the vast range of possible linkages between the different moieties. Whereas, proton nuclear magnetic resonance (^1^H-NMR) spectroscopy is key to proof the structural identity and purity of organic molecules, it is of little use for CBCs. ^1^H-NMR spectra can in principle be used to determine the molar ratios of the different components in a CBC (Lee et al., 2018; Park et al., 2019; Park S. H. et al., 2020; Tseng et al., 2022), but the reasonably large molar mass and strong aggregation tendency of push-pull copolymers often cause severe peak broadening, thereby masking all structural information. Even for a highly soluble CBC, the polymer composition is likely too complex to unravel by NMR and the sensitivity is too low to elucidate the often nuanced effect of structural defects like homocoupling. Gel permeation chromatography (GPC) enables to monitor shifts in molar mass and dispersity when adding the second block, but this does not exclude that the first block continues to grow individually (Li et al., 2021, 2022; Wu et al., 2021, 2022; Cheng et al., 2023; Liu B. et al., 2023; Zheng et al., 2023). Moreover, no structural information can be deduced from GPC measurements. UV-Vis-NIR absorption spectra can indicate that the individual D and A polymers are indeed present in the final polymer mixture but do not provide any information on the connectivity of the blocks. A physical mixture of the separate D and A polymers would give a very similar absorption profile. In present SC-OSC literature, energy-dispersive X-ray spectroscopy (EDX) and/or X-ray photoelectron spectroscopy (XPS) are used to evaluate the molar ratios of the blocks (Park et al., 2019; Li et al., 2021; Wu et al., 2021; Cheng et al., 2023; Liu B. et al., 2023). However, also here, no real structural information can be deducted from these molar ratios. Even a combination of all of the above-mentioned techniques falls short in providing real structural insights. Considering, on top of that, homocoupling defects can be quite prominent in state-of-the-art push-pull OPV polymers and may have a strong impact on final solar cell performance (Smeets et al., 2023), it is clear that additional efforts are required to unravel the structure of CBCs and to investigate how this structural variability influences the final SC-OSC efficiency and stability.

## 3 Pathways to enhanced control over the CBC structure

From the above, it is obvious that further development in field of SC-OSCs is hampered by the lack of control over the CBC structure as well as the troublesome characterization. Both aspects severely hinder the establishment of structure-property-device relations. In this perspective, we propose a stepwise approach to tackle this challenge, starting from the more controlled synthesis and detailed analysis of the individual blocks before gradually moving to the final CBCs.

Unfortunately, the Stille cross-coupling polymerization—the currently most applied procedure for push-pull polymer and CBC synthesis—is not the most “controlled” of all polymerization methods. Although advanced catalyst-transfer polymerizations would surely afford better control over the block length and end-groups, and could even allow a “living” polymerization (and on top of that would avoid organotin toxicity issues) (Woods et al., 2020; Luscombe et al., 2022; Xu et al., 2023), the complex alternating push-pull motifs of the state-of-the-art OPV polymers cannot be realized by the controlled conjugated polymer synthesis procedures available to date. For this reason, we focus on Stille polymerization here. This does not prevent, however, that the gathered knowledge can be translated to, for instance, direct arylation polymerization protocols at a later stage (Nakabayashi, 2018; Chua et al., 2021).

In terms of polymer analysis, routine investigation with matrix-assisted laser desorption-ionization—time of flight mass spectrometry (MALDI-ToF MS) would be a very welcome addition to the SC-OSC field. The gentle ionization ensures minimal to no fragmentation. As a result, one can assume that the masses and isotope patterns correspond to complete chains. MALDI-ToF MS hence provides information on the type and number of monomers present in each oligomer/polymer chain as well as the end-groups. Over the past years, our group has successfully applied MALDI-ToF MS on a wide range of alternating conjugated polymers, in particular for the identification of homocoupling defects and polymer end-groups (Vangerven et al., 2015; Pirotte et al., 2018; Smeets et al., 2023; Vanderspikken et al., 2023). However, MALDI-ToF MS also has its limitations. First of all, the technique is most sensitive to low-molar-mass species. This results in spectra preferentially showing the lower-molar-mass components in a mixture. Secondly, the ionization tendency of varying species can be quite different depending on their composition, end-groups, and the matrix used, even within the same mass range, and no absolute quantification can hence be done. Moreover, even though the number of the different monomers present can be deduced, there is no information on their connectivity. Finally, detailed manual assignment of all species—by recognizing recurring patterns, calculating the remaining mass, and subsequently aligning it with feasible end-groups—is not trivial and requires expert insights on the Stille cycle and its possible side reactions. Nevertheless, MALDI-ToF MS analysis can provide valuable information on the CBC backbone structure that cannot be retrieved by the currently applied characterization methods. So far, however, the use of MALDI-ToF MS has not been embraced by the SC-OSC community. Only in the recent report by Phan et al. (2022), a (two-pot synthesis) CBC product was subjected to MALDI-ToF MS analysis. The number-average molar mass (*M*_n_) was in line with the GPC results but no real assignment of species was performed. We call on the field to routinely apply MALDI-ToF MS to get a more realistic picture of the different species present in their CBC products, e.g., sole A or D chains, oligomers, block copolymers, active or terminated chains, etc.

**Figure 2:**
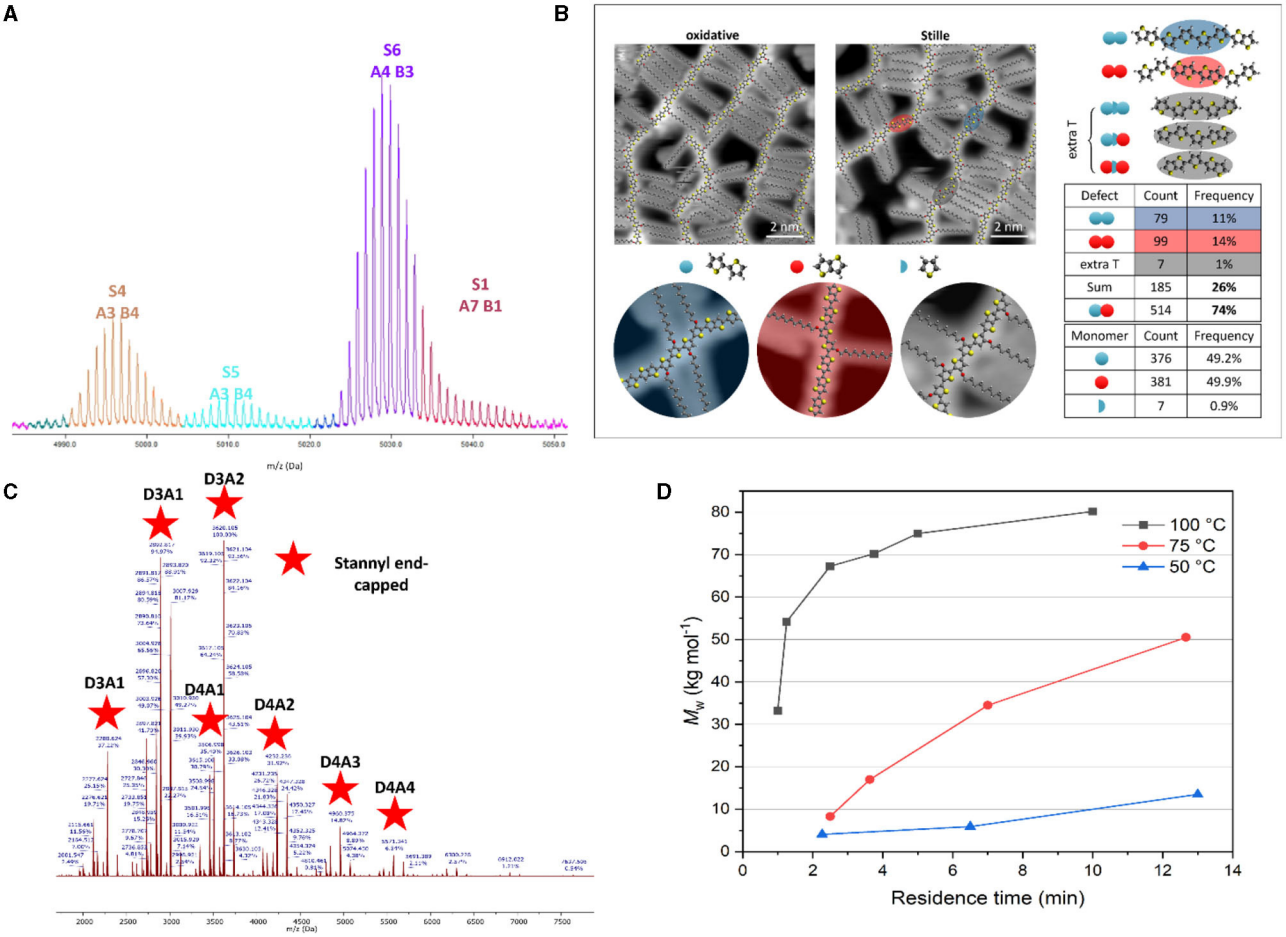
**(A)** MALDI-ToF MS spectrum excerpt for D18, annotated by commercial software. **(B)** STM imaging and defect quantification for alkoxylated PBTTT (reprinted with permission from Vanderspikken et al., 2023). **(C)** MALDI-ToF mass spectrum for 2TC-TT-BDTFT (16 h reaction at 100°C; see Supplementary material for details), with assignment of the stannyl end-capped species (D, donor monomer; A, acceptor monomer). **(D)** Illustration of molar mass tailoring of conjugated polymers by droplet-flow, illustrated for PM6 (reprinted with permission from Smeets et al., 2023).

For a prototypical CBC structure (Figure 1D), four different monomers are copolymerized. This leads to a large variety of possible products that risks to become a difficult puzzle to analyse. To this end, automated MS signal assignment and statistical analysis software specifically for conjugated (block) copolymers would be very welcome. While specialized (commercial and open source) software for copolymer analysis does exist, the application thereof on mass spectra of conjugated copolymers remains difficult. For example, even though commercial software can annotate mass spectra in an intuitive way, it fails at correctly dividing the relative abundance of overlapping identified species, and thus derived copolymer statistics are incorrect. An illustration of this problem is given in Figure 2A. In the mass spectrum of the alternating conjugated polymer D18, the software incorrectly identifies the weakly intense tail-end of the S6 signal to the (unlikely) highly homocoupled species S1. Since the amount of plausible species gets much larger when analysing CBCs, this issue is expected to increase in severity. Moreover, current tools cannot be used for polymer systems containing more than two monomers, such as CBCs. Therefore, the development of software which can automatically annotate and process the intricate mass spectra of CBCs through deconvolution of overlapping signals is highly desirable.

Ultrahigh vacuum scanning tunneling microscopy (UHV-STM) presents a very powerful analysis technique for conjugated polymers, complementary to MALDI-ToF MS, since it is able to visualize the exact polymer sequence and to identify and even quantify structural defects (Warr et al., 2018; Ponder et al., 2021). This was recently applied for the analysis of homocoupling in an alkoxylated version of the benchmark semi-crystalline polymer PBTTT (Figure 2B) (Vanderspikken et al., 2023). If this could be used for CBCs, it would obviously be very helpful to characterize them and acquire deeper insights on the CBC composition and polymerization mechanism. However, the STM technique is not widely available, the statistical analysis is labor-intensive, and end-group identification remains difficult.

To achieve more insights on the CBC polymerization, a stepwise two-pot synthesis strategy seems most appropriate. This allows detailed characterization of the separate D and A blocks, e.g., by MALDI-ToF MS (while even NMR could be useful if the chains are small enough). At present, a 1:1 ratio of the stannylated and halogenated monomers is commonly used for the synthesis of the individual D and A blocks and the reaction time is kept short to ensure that enough chain ends are still “active”. However, a (slight) excess of one of the monomers would afford a more homogeneous mixture of oligomers with either predominant stannyl or halogen end-groups. Polymerization of these separate blocks with complementary end-groups—which can individually be analyzed by MALDI-ToF MS—should be more selectively and should hence result in a better defined CBC structure. We have performed a quick test to illustrate this approach. A small excess of a distannylated benzo[1,2-*b*:4,5-*b*']dithiophene (BDT) monomer (1.3 equiv) afforded short D oligomer chains (*M*_n_ = 7.2 kDa, Ð* = 1.5) of 2TC-TT-BDTFT (Chen et al., 2018) that are mostly stannyl end-capped (Figure 2C). It has to be noted though that BDT homocoupling can be seen as well. Such homocoupling defects have been shown to have a (mostly negative) impact on final solar cell performance and may even mask the true potential of a material, and hence have to be avoided (Hendriks et al., 2014; Vangerven et al., 2015; Lombeck et al., 2016; Pirotte et al., 2018; Ma et al., 2022; Smeets et al., 2023; Vanderspikken et al., 2023).

Our group has embraced continuous flow chemistry to achieve enhanced control over the structure of push-pull conjugated polymers and minimize batch-to-batch variations (Pirotte et al., 2015; Beckers et al., 2020, 2022; Smeets et al., 2023). Besides the generally accepted advantages in terms of safety, scalability, speed, and reaction optimization, flow chemistry allows to tailor the molar mass of state-of-the-art OPV polymers (Smeets et al., 2023). It is known that a reasonably high molar mass of the D polymer (while keeping it soluble) is one of the main requirements to get optimally performing organic solar cells (Chu et al., 2012; Bartelt et al., 2014; Lee et al., 2014; Gasparini et al., 2015; Li et al., 2017). Droplet-flow chemistry avoids diffusion of the polymer product into the solvent stream, thereby allowing the synthesis of conjugated polymers with an optimal and reproducible molar mass. When applied to PM6, combined with an optimized Stille catalytic system (Ma et al., 2022), our droplet-flow protocol afforded this D polymer with a predefined molar mass (Figure 2D) and a strongly reduced homocoupling content. This approach is thus very attractive for CBC synthesis, in particular since PM6 (or a derivative thereof) is applied as the D part for some of the best-performing CBCs to date. Bi et al. (2023) investigated the impact of the molar mass of the polymer acceptor in PM6:PYIT all-polymer solar cells. Their findings revealed a substantial influence of the A polymer's length on the aggregation behavior and overall performance of the polymer blend. Consequently, we propose to control the size of both the D and A blocks by separately preparing them in continuous flow streams and then combine these further on. In a CBC optimization context, the flow chemistry approach emerges as the go-to technique, since it enables swift optimization of various reaction parameters like temperature, residence time, and concentration.

As a final note, we would like to mention that the currently best-performing CBCs have rather complex structures. As synthetic complexity is part of the i-FOM metric, it would be good to not only focus on PM6, PYIT, and related high-efficiency D and A polymers but rather to start introducing “simpler”, more industrially relevant materials [e.g., PTQ-10 as the D part (Szymanski et al., 2020)], preferably even containing bio-based building blocks.

## 4 Conclusions

Ever since the emergence of the first organic-based solar cells, scientists have struggled with the necessity to combine two different materials in the photoactive layer to realize efficient exciton splitting. Although the power conversion efficiency has strongly improved over the past three decades, the intimate bulk heterojunction nanomorphology and its (in)stability over time remain important points of attention. In last years, however, remarkable steps have been taken to realize the old dream of “single-component” organic solar cells. The spectacular surge in efficiency for both polymer:NFA and all-polymer solar cells has motivated material chemists to combine the top donor and acceptor structures in single “conjugated block copolymer” chains. Within a short period of time, impressive solar cell efficiencies (up to 15%) and enhanced stabilities have been reported for these novel materials. Nevertheless, we are convinced that the best is yet to come for single-component organic photovoltaics. In this perspective, we listed the different aspects that—in our opinion—can still be improved. We pointed to the lack of structural control and troublesome characterization of the state-of-the-art CBCs and made different suggestions to enhance the chemical precision, facilitate analysis, and improve reproducibility, all of this with the overarching aim to establish rational structure-device relationships as an additional leverage for SC-OSCs and OPV in general. As a last recommendation, we highlighted the importance of “synthetic simplicity” for industrial valorization. Combined with simple ink formation and large-area printing options, these might be the final pieces to convince SMEs and potential investors that the future is really organic. Finally, the knowledge gained on single-component organic semiconductors will not only benefit the solar cell field. Cross-over to other emerging green energy technologies, such as solar-to-hydrogen conversion, is on the horizon as well (Kosco et al., 2022).

## Data availability statement

The raw data supporting the conclusions of this article will be made available by the authors, without undue reservation.

## Author contributions

DT: Writing—original draft, Writing—review & editing, Investigation. SS: Investigation, Writing—review & editing. WM: Writing—review & editing, Conceptualization, Funding acquisition, Writing—original draft, Supervision.
