# Supplementary material for: Single-component organic solar cells—Perspective on the importance of chemical precision in conjugated block copolymers
Source: Front Chem. 2023 Dec 12;11:1326131. doi: 10.3389/fchem.2023.1326131 (PMC11061845; doi:10.3389/fchem.2023.1326131)
Supplement: Supplementary file 1 [file Presentation_1.pdf]

Supplementary Material

Supplementary Material

Methods section

Synthesis of 2TC-TT-BDTFT

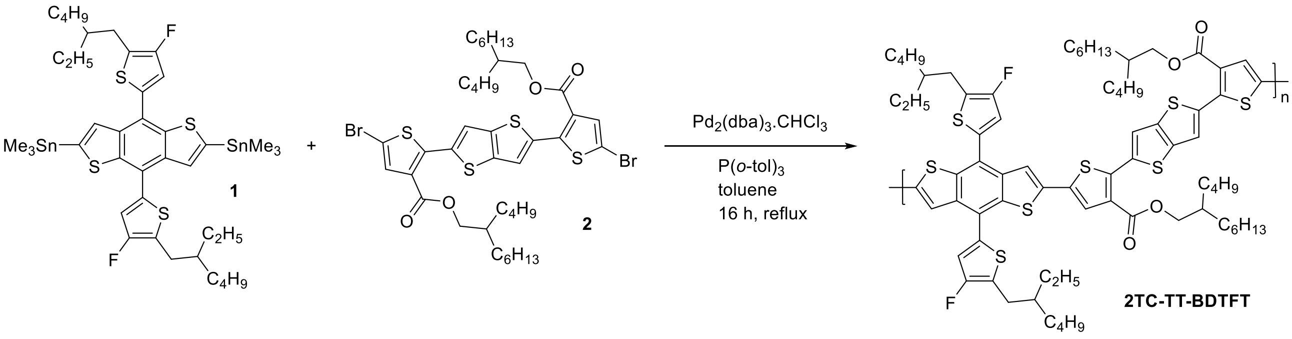

To {4,8-bis[5-(2-etylhexyl)-4-fluorothiophen-2-yl]benzo[1,2-*b*:4,5-*b*']dithiophene-2,6-diyl}bis(tri-methylstannane) (**1**) (94 mg, 0.1 mmol, 1.3 equiv.) and bis(2-butyloctyl)-2,2'-(thieno[3,2-*b*]thiophene-2,5-diyl)bis(5-bromothiophene-3-carboxylate) (**2**) (89 mg, 0.77 mmol, 1 equiv) in a dried schlenk tube, $Pd_2(dba)_3{\cdot}CHCl_3$ (3.1 mg, 0.003 mmol, 0.039 equiv) and P(*o*-tol)$_3$ (3.7 mg, 0.12 mmol, 0.15 equiv) were added and the reaction mixture was put under argon atmosphere. Subsequently, toluene (2 mL) was added. The reaction vessel was subjected 5x to a vacuum-Ar cycle and heated at 110 °C for 16 h. The reaction mixture was then added to MeOH (100 mL) and the resulting precipitate was transferred into an extraction thimble. Soxhlet extraction was performed in the following order: methanol, acetone, *n*-hexane, and dichloromethane. Extractions were always done until the solvent in the extraction chamber was colorless. Finally, the dichloromethane fraction was precipitated, yielding 62 mg (60%) of a red solid. SEC (1,2,4-TCB,160 °C, PS standards): M_n = 7.2 kDa, M_w = 11.1 kDa, M_p = 8.6 kDa, *Đ* = 1.5.

Matrix-assisted laser desorption-ionization - time of flight mass spectrometry (MALDI-ToF MS)

Mass spectra were recorded on a Bruker UltrafleXtremeTM MALDI-ToF/ToF system. Approximately 1 µL of the matrix solution (25 mg mL^{-1} *trans*-2-[3-(4-*tert*-butylphenyl)-2-methyl-2-propenylidene]-malononitrile (DTCB) in $CHCl_3$) was spotted onto an MTP Anchorchip 600/384 MALDI plate. The spot was allowed to dry and 1 µL of the analyte solution (10 mg mL^{-1} in $CHCl_3$) was spotted on top of the matrix.

Gel permeation chromatography (GPC)

Polymer molar mass distributions were estimated by SEC at 160 °C on an Agilent 1260 Infinity II high-temperature gel permeation chromatography (GPC) system using a PL-GEL 10 µm MIXED-B column with 1,2,4-trichlorobenzene as the eluent and using polystyrene (PS) internal standards.

Supplementary Figures and Tables

Supplementary Table 1. Evolution of the power conversion efficiency of SC-OSCs based on CBCs (data used to produce Figure 1a).

Publication Year	PCE SC-OSCs (%)	Reference
2013	3.1	(1)
2015	2.24	(2)
2015	0.55 – 0.95	(3)
2016	1.00	(4)
2016	0.22	(5)
2017	0.36 – 1.54	(6)
2018	3.87	(7)
2019	5.28	(8)
2020	6.43	(9)
2021	11.32	(10)
2021	4.20 – 8.64	(11)
2022	14.88	(12)
2022	10.51	(13)
2022	10.55	(14)
2022	4.35	(15)
2023	14.30	(16)
2023	13.73	(17)
2023	13.40	(18)
2023	13.40	(19)

Supplementary Table 1. Data for the efficiency and stability comparison of CBC-based SC-OSCs and binary all-polymer solar cells (Figure 1b-c).

Reference	PCE SC-OSCs (%)	PCE all-polymer solar cells (%)	Temperature (°C)[a]	Duration (h)[a]	Residual PCE SC-OSCs (normalized)	Residual PCE all-polymer solar cells (normalized)
(10)	11.32	14.57	ambient	240	0.85	0.71
(11)	8.64	12.2	80	1000	0.80	0.64
(12)	14.88	14.57	85	500	0.87	0.66
(14)	10.55	8.24	120	400	0.90	0.80
(17)	13.73	13.09	85	240	0.67	0.54
(18)	13.4	12.26	ambient	480	0.91	0.88

[a] Temperature and duration of the (thermal) treatment to induce solar cell degradation.

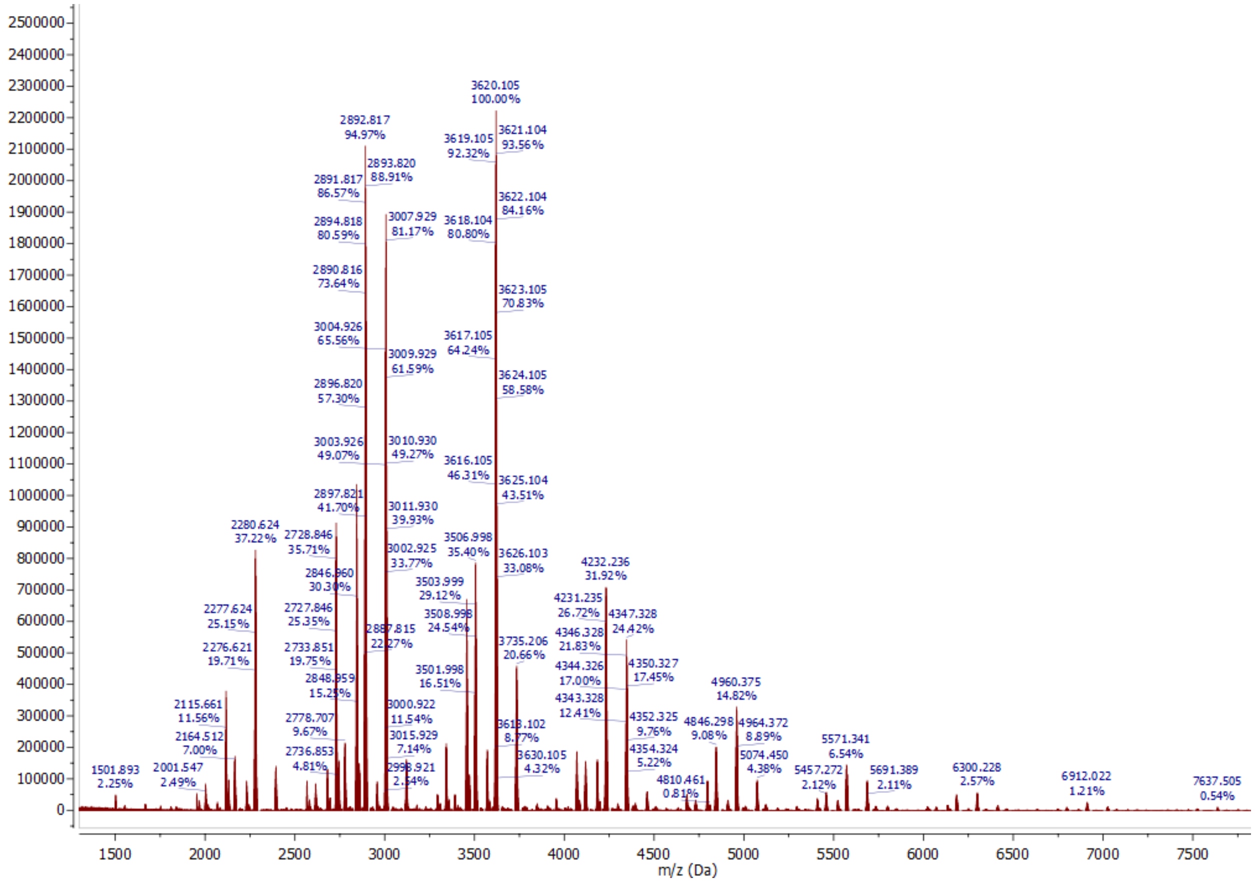

Supplementary Figure 1. MALDI-ToF mass spectrum of 2TC-TT-BDTFT.

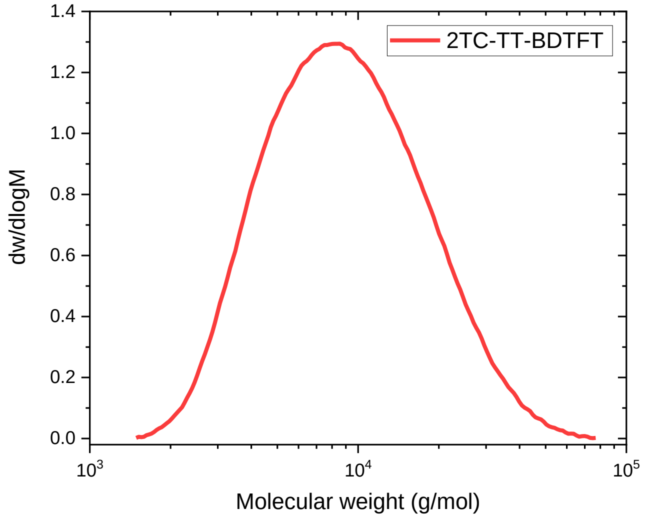

Supplementary Figure 2. GPC trace for 2TC-TT-BDTFT.

References

1. Guo C, Lin YH, Witman MD, Smith KA, Wang C, Hexemer A, et al. Conjugated block copolymer photovoltaics with near 3% efficiency through microphase separation. Nano Lett. 2013;13(6):2957-63.

2. Mok JW, Lin Y-H, Yager KG, Mohite AD, Nie W, Darling SB, et al. Linking Group Influences Charge Separation and Recombination in All-Conjugated Block Copolymer Photovoltaics. Advanced Functional Materials. 2015;25(35):5578-85.

3. Lombeck F, Komber H, Sepe A, Friend RH, Sommer M. Enhancing Phase Separation and Photovoltaic Performance of All-Conjugated Donor–Acceptor Block Copolymers with Semifluorinated Alkyl Side Chains. Macromolecules. 2015;48(21):7851-60.

4. Wang SF, Yang QQ, Tao YT, Guo Y, Yang J, Liu YA, et al. Fully conjugated block copolymers for single-component solar cells: synthesis, purification, and characterization. New Journal of Chemistry. 2016;40(2):1825-33.

5. Li Y, Shetye K, Baral K, Jin L, Oster JD, Zhu D-M, et al. Main-chain polyoxometalate-containing donor–acceptor conjugated copolymers: synthesis, characterization, morphological studies and applications in single-component photovoltaic cells. RSC Advances. 2016;6(36):29909-19.

6. Lee DH, Lee JH, Kim HJ, Choi S, Park GE, Cho MJ, et al. (D)n–σ–(A)m type partially conjugated block copolymer and its performance in single-component polymer solar cells. Journal of Materials Chemistry A. 2017;5(20):9745-51.

7. Lee JH, Park CG, Kim A, Kim HJ, Kim Y, Park S, et al. High-Performance Polymer Solar Cell with Single Active Material of Fully Conjugated Block Copolymer Composed of Wide-Band gap Donor and Narrow-Band gap Acceptor Blocks. ACS Appl Mater Interfaces. 2018;10(22):18974-83.

8. Park CG, Park SH, Kim Y, Nguyen TL, Woo HY, Kang H, et al. Facile one-pot polymerization of a fully conjugated donor–acceptor block copolymer and its application in efficient single component polymer solar cells. Journal of Materials Chemistry A. 2019;7(37):21280-9.

9. Park SH, Kim Y, Kwon NY, Lee YW, Woo HY, Chae WS, et al. Significantly Improved Morphology and Efficiency of Nonhalogenated Solvent-Processed Solar Cells Derived from a Conjugated Donor-Acceptor Block Copolymer. Adv Sci (Weinh). 2020;7(4):1902470.

10. Wu Y, Guo J, Wang W, Chen Z, Chen Z, Sun R, et al. A conjugated donor-acceptor block copolymer enables over 11% efficiency for single-component polymer solar cells. Joule. 2021;5(7):1800-15.

11. Li S, Yuan X, Zhang Q, Li B, Li Y, Sun J, et al. Narrow-Bandgap Single-Component Polymer Solar Cells with Approaching 9% Efficiency. Adv Mater. 2021;33(32):e2101295.

12. Wu Y, Fan Q, Fan B, Qi F, Wu Z, Lin FR, et al. Non-Fullerene Acceptor Doped Block Copolymer for Efficient and Stable Organic Solar Cells. ACS Energy Letters. 2022;7(7):2196-202.

13. Li S, Li B, Yang X, Wei H, Wu Z, Li Y, et al. Regioselectivity control of block copolymers for high-performance single-material organic solar cells. Journal of Materials Chemistry A. 2022;10(24):12997-3004.

14. Phan TN, Lee JW, Oh ES, Lee S, Lee C, Kim TS, et al. Efficient and Nonhalogenated Solvent-Processed Organic Solar Cells Enabled by Conjugated Donor-Acceptor Block Copolymers Containing the Same Benzodithiophene Unit. ACS Appl Mater Interfaces. 2022;14(51):57070-81.

15. Tseng YC, Kato A, Chang JF, Chen WC, Higashihara T, Chueh CC. Impact of the segment ratio on a donor-acceptor all-conjugated block copolymer in single-component organic solar cells. Nanoscale. 2022;14(14):5472-81.

16. Liu B, Sun H, Lee JW, Jiang Z, Qiao J, Wang J, et al. Efficient and stable organic solar cells enabled by multicomponent photoactive layer based on one-pot polymerization. Nat Commun. 2023;14(1):967.

17. Zheng Y, Wu Y, Chen Z, Xia X, Li Y, Wu Q, et al. Unraveling the device performance differences between bulk-heterojunction and single-component polymer solar cells. Journal of Materials Chemistry A. 2023;11(16):8961-71.

18. Cheng Y, Mao Q, Zhou C, Huang X, Liu J, Deng J, et al. Regulating the Sequence Structure of Conjugated Block Copolymers Enables Large-Area Single-Component Organic Solar Cells with High Efficiency and Stability. Angewandte Chemie. 2023; 10.1002/anie.202308267.

19. Chen M, Wang S, Gao Y, Shao Y, Xu L, Wu Y, Wang Y, Ashraf R, Ponomarenko S, Luponosov Y, Min j, et al. The application of chlorine substituted conjugated block copolymers in the single-component organic solar cells. Giant. 2023; 10.1016/j.giant.2023.100191.
